# Supplementary material for: First Trimester Maternal Homocysteine and Embryonic and Fetal Growth: The Rotterdam Periconception Cohort
Source: Nutrients. 2022 Mar 8;14(6):1129. doi: 10.3390/nu14061129 (PMC8953595; doi:10.3390/nu14061129)
Supplement: Supplementary file 1 [file nutrients-14-01129-s001.zip › nutrients-1546547-supplementary.pdf]

Table S1. Comparison between cases of reference range homocysteine and hyperhomocysteinemia in the starting population.

**Characteristics**	**tHcy < 15 µmol/L (n= 2299)**	**Hyperhomocsyteinemia > 15 µmol/L (n= 13)**	**P-value**
Age[a], mean ± SD	32.1± 4.6	28.9 ± 6.0	0.86
Ethnicity, n (%)			0.839
Dutch	1629 (81)	9 (75)	
Western, other	101 (5)	1 (8)	
Non-western	292 (14)	2 (2)	
Education level, n (%)			**0.035**
Low	166 (8)	3 (25)	
Intermediate	714 (36)	6 (50)	
High	1122 (56)	3 (25)	
BMI[b], median (IQR)	25.2 (22.5-29.0)	25.4 (22.9-31.7)	0.628
Nulliparous, n (%)	729 (36)	3 (25)	0.554
Mode of conception, n (%)			0.194
Natural	1599 (65)	11 (85)	
IVF/ICSI	700 (30)	2 (15)	
Alcohol use, n (%)	620 (31)	1 (8)	0.119
Smoking, n (%)	301 (15)	3 (25)	0.407
Folic acid supplement use, n (%)	1962 (97)	11 (92)	0.269
Caffeine use, n (%)			0.324
None	908 (45)	8 (67)	
Moderate	816 (40)	3 (25)	
High	292 (14)	1 (8)	
**Biomarkers***	**tHcy < 15 µmol/L (n= 2299)**	**Hyperhomocsyteinemia > 15 µmol/L (n= 13)**	**P-value**
Serum folate[c], median (IQR)	37.2 (28.5-43.5)	11.5 (10.5-45.15)	**0.025**
Vitamin B_{12}[d], median (IQR)	297.0 (226-375)	207.0 (175-327)	0.242
Paternal tHcy[e], median (IQR)	11.5 (9.9-13.7)	12.45 (11.53-13.8)	0.409
Paternal serum folate[c], median (IQR)	17.7 (14-22.4)	17.45 (13.63-25.4)	0.914
Paternal vitamin B_{12}[d], median (IQR)	340 (268-424.5)	475.0 (325.75-743.5)	0.064

[a] years, [b] kg/m^2, [c] nmol/L, [d] pmol/l, [e] µmol/L. * Subgroup from November 2010 to December 2016. Data on alcohol use, smoking, folic acid supplementation and caffeine use were collected during the periconception period. Parametric data is represented as mean ± SD, whereas non-parametric data is represented as median (IQR). Abbreviations: BMI, body mass index; IUI, intrauterine insemination; IVF, in vitro fertilization; ICSI, intracytoplasmic sperm injection; SD, standard deviation; IQR, interquartile range. P-value <0.05 represented in bold.

Table S2. Maternal baseline characteristics of the included and excluded population.

Characteristics	Included population (n = 1060)	Missing values, n (%)	Excluded population (n = 1252)	Missing values, n (%)	P-value
Age[a], mean ± SD	32.4 ± 4.4	0 (0)	31.8 ± 4.8	0 (0)	**0.001**
Ethnicity, n (%)		48 (5)		230 (18)	0.273
Dutch	814 (80)		824 (81)		
Western, other	58 (6)		44 (4)		
Non-western	140 (14)		154 (15)		
Education level, n (%)		53 (5)		245 (20)	**0.048**
Low	71 (7)		98 (10)		
Intermediate	353 (35)		367 (36)		
High	583 (58)		542 (54)		
BMI[b], median (IQR)	24.5 (22.1-27.9)	28 (3)	25.8 (22.9-29.7)	45 (4)	**0.000**
Nulliparous, n (%)	380 (37)	45 (4)	352 (35)	237 (19)	0.212
Mode of conception, n (%)		0 (0)		0 (0)	**0.000**
Natural	555 (52)		1055 (84)		
IVF/ICSI	505 (48)		197 (16)		
Alcohol use, n (%)	307 (30)	49 (5)	314 (31)	253 (20)	0.629
Smoking, n (%)	143 (14)	47 (5)	161 (16)	254 (20)	0.214
Folic acid supplement use, n (%)	1004 (99)	46 (4)	969 (96)	241 (19)	**0.000**
Caffeine use, n (%)		45 (4)		239 (19)	0.563
None	456 (45)		460 (45)		
Moderate	404 (40)		415 (41)		
High	155 (15)		138 (14)		
tHcy[e], mean ± SD	6.4 ± 1.6	0 (0)	6.5 ± 2.7	0 (0)	0.433
**Biomarkers***	**Included population (n = 682)**	**Missing values, n (%)**	**Excluded population (n = 1061)**	**Missing values, n (%)**	**P-value**
tHcy[e], median (IQR)	6.1 (5.3-7.1)	0 (0)	5.9 (5.0-7.2)	0 (0)	0.208
Serum folate[c], median (IQR)	39.7 (33.2-45.4)	27 (4)	35.2 (24.1-42.3)	54 (5)	**0.000**
Vitamin B_{12}[d], median (IQR)	316.0 (246.0-402.0)	7 (1)	281.0 (215.0-359.0)	16 (2)	**0.000**
Paternal tHcy[e], median (IQR)	11.6 (9.9-13.6)	132 (19)	11.2 (9.8-13.3)	256 (24)	0.128
Paternal serum folate[c], median (IQR)	18.1 (14.4-22.6)	133 (20)	17.5 (13.7-22.2)	257 (24)	0.267
Paternal vitamin B_{12}[d], median (IQR)	337.0 (265.0-416.5)	133 (20)	341.5 (271.0-432.5)	251 (24)	0.130

[a]years, [b]kg/m², [c]nmol/L, [d]pmol/l, [e]µmol/L. * Subgroup from November 2010 to December 2016. Alcohol use, smoking, folic acid supplementation and caffeine use were measured during the periconception period. Parametric data is represented as mean ± SD, whereas non-parametric data is represented as median (IQR). Abbreviations: BMI, body mass index; IUI, intrauterine insemination; IVF, in vitro fertilization; ICSI, intracytoplasmic sperm injection; SD, standard deviation; IQR, interquartile range. P-value <0.05 represented in bold.

Table S3. Association between maternal homocysteine, CRL and EV at 7, 9 and 11 weeks of gestation.

		Model 1		*Model 2*	
**Included population**					
		**CRL**			
		Beta (95%CI) √mm	P-value	Beta (95%CI) √mm	P-value
7 weeks	n= 502	**-0.029 (-0.047,-0.011)**	**0.002**	**-0.025 (-0.046,-0.005)**	**0.016**
9 weeks	n= 778	**-0.019 (-0.032,-0.006)**	**0.005**	**-0.021 (-0.035,-0.007)**	**0.003**
11 weeks	n= 830	**-0.028 (-0.044,-0.013)**	**<0.001**	**-0.013 (-0.022,-0.004)**	**0.006**
		**EV**			
		Beta (95%CI) $\sqrt[3]{}$cm	P-value	Beta (95%CI) $\sqrt[3]{}$cm	P-value
7 weeks	n= 475	**-0.011 (-0.019,-0.003)**	**0.006**	**-0.010 (-0.019,-0.001)**	**0.024**
9 weeks	n= 740	**-0.009 (-0.015,-0.002)**	**0.007**	**-0.009 (-0.015,-0.002)**	**0.010**
11 weeks	n= 722	**-0.013 (-0.022,-0.004)**	**0.006**	**-0.010 (-0.020,-0.001)**	**0.035**
**Natural pregnancies**					
		**CRL**			
		Beta (95%CI) √mm	P-value	Beta (95%CI) √mm	P-value
7 weeks	n= 234	**-0.038 (-0.070,-0.006)**	**0.022**	-0.032 (-0.069,0.005)	0.089
9 weeks	n= 370	-0.018 (-0.039,0.003)	0.091	**-0.023 (-0.046,-0.001)**	**0.041**
11 weeks	n= 426	-0.021 (-0.045,0.003)	0.085	-0.017 (-0.044,0.009)	0.204
		**EV**			
		Beta (95%CI) $\sqrt[3]{}$cm	P-value	Beta (95%CI) $\sqrt[3]{}$cm	P-value
7 weeks	n= 219	**-0.017 (-0.030,-0.004)**	**0.011**	-0.015 (-0.030,0.000)	0.054
9 weeks	n= 348	**-0.010 (-0.020,0.000)**	**0.047**	**-0.012 (-0.022,-0.001)**	**0.029**
11 weeks	n= 377	-0.010 (-0.023,0.004)	0.178	-0.005 (-0.020,0.009)	0.468
**IVF/ICSI pregnancies, frozen embryo transfer**					
		**CRL**			
		Beta (95%CI) √mm	P-value	Beta (95%CI) √mm	P-value
7 weeks	n= 84	-0.039 (-0.079,0.002)	0.061	**-0.042 (-0.085,0.000)**	**0.049**
9 weeks	n= 135	**-0.035 (-0.068,-0.002)**	**0.035**	**-0.038 (-0.073,-0.002)**	**0.038**
11 weeks	n= 125	**-0.041 (-0.075,-0.008)**	**0.017**	**-0.047 (-0.083,-0.010)**	**0.014**
		**EV**			
		Beta (95%CI) $\sqrt[3]{}$cm	P-value	Beta (95%CI) $\sqrt[3]{}$cm	P-value
7 weeks	n= 84	-0.015 (-0.039,0.008)	0.194	-0.017 (-0.044,0.009)	0.190
9 weeks	n= 130	**-0.016 (-0.032,0.000)**	**0.047**	**-0.018 (-0.035,-0.001)**	**0.035**
11 weeks	n= 110	**-0.026 (-0.047,-0.004)**	**0.018**	-0.022 (-0.045,0.001)	0.060
**IVF/ICSI pregnancies, fresh embryo transfer**					

		CRL			
		Beta (95%CI) √mm	P-value	Beta (95%CI) √mm	P-value
7 weeks	n= 184	-0.11 (-0.031,0.008)	0.253	-0.009 (-0.032,0.013)	0.400
9 weeks	n= 273	-0.013 (-0.031,0.005)	0.163	-0.009 (-0.029,0.011)	0.364
11 weeks	n= 279	**-0.031 (-0.050,-0.014)**	**0.001**	**-0.033 (-0.052,-0.013)**	**0.001**
		**EV**			
		Beta (95%CI) $\sqrt[3]{}$cm	P-value	Beta (95%CI) $\sqrt[3]{}$cm	P-value
7 weeks	n= 172	0.001 (-0.007,0.008)	0.846	0.001 (-0.008,0.009)	0.886
9 weeks	n= 262	0.000 (-0.008,0.008)	0.937	0.001 (-0.008,0.009)	0.906
11 weeks	n= 235	**-0.012 (-0.023,0.000)**	**0.048**	-0.010 (-0.022,0.001)	0.081

CRL= crown-rump length, EV= embryonic volume. Model 1: adjusted for GA, Model 2: adjusted for GA, conception mode (included population only), parity, smoking, folic acid supplement use, alcohol, BMI, age and fetal sex. N number was too small to analyze CRL and EV at 13 weeks of gestation. P-value <0.05 represented in bold.

Table S4. Association between maternal homocysteine and longitudinal first trimester embryonic head measurements in the included population (n= 118), natural pregnancies (n= 71) and IVF/ICSI pregnancies (n= 47).

	Model 1		*Model 2*	
	**BPD**			
	Beta (95%CI) mm	P-value	Beta (95%CI) mm	P-value
Included population n= 104	0.080 (-0.314, 0.154)	0.496	-0.079 (-0.321, 0.163)	0.518
Natural pregnancies n= 65	-0.153 (-0.482, 0.176)	0.355	-0.142 (-0.471, 0.187)	0.389
IVF/ICSI pregnancies n= 39	0.023 (-0.257, 0.303)	0.866	-0.072 (-0.401, 0.256)	0.653
	**OFD**			
	Beta (95%CI) mm	P-value	Beta (95%CI) mm	P-value
Included population n= 104	-0.083 (-0.328, 0.161)	0.500	-0.029 (-0.279, 0.219)	0.812
Natural pregnancies n= 65	-0.144 (-0.482, 0.193)	0.394	-0.079 (-0.400, 0.241	0.621
IVF/ICSI pregnancies n= 39	-0.046 (-0.371, 0.278)	0.774	-0.117 (-0.497, 0.263)	0.531
	**HV**			
	Beta (95%CI) cm^3	P-value	Beta (95%CI) cm^3	P-value
Included population n= 104	-0.017 (-0.172, 0.136)	0.818	-0.010 (-0.167, 0.146)	0.898
Natural pregnancies n= 65	-0.08 (-0.306, 0.141)	0.464	-0.071 (-0.289, 0.148)	0.521
IVF/ICSI pregnancies n= 39	0.039 (-0.102, 0.181)	0.575	0.003 (-0.156, 0.163)	0.966
	**HC**			
	Beta (95%CI) mm	P-value	Beta (95%CI) mm	P-value
Included population n= 104	-0.251 (-0.969, 0.467)	0.488	-0.159 (-0.897, 0.578)	0.667
Natural pregnancies n= 65	-0.464 (-1.469, 0.540)	0.357	0.340 (-1.319, 0.641)	0.489
IVF/ICSI pregnancies n= 39	-0.050 (-0.927, 0.825)	0.906	-0.312 (-1.335, 0.709)	0.533
	**TCD**			
	Beta (95%CI) mm	P-value	Beta (95%CI) mm	P-value
Included population n= 81	-0.027 (-0.129, 0.074)	0.589	-0.002 (-0.111, 0.108)	0.974
Natural pregnancies n= 48	-0.128 (-0.293, 0.035)	0.121	-0.047 (-0.219, 0.125)	0.581
IVF/ICSI pregnancies n= 33	0.030 (-0.079, 0.139)	0.575	0.001 (-0.116, 0.118)	0.983

BPD= biparietal diameter, HC= head circumference, HV= head volume, OFD= occipitofrontal diameter, TCD= transcerebellar diameter. Model 1: adjusted for GA, Model 2: adjusted for conception mode (included population only), parity, smoking, folic acid supplement use, alcohol, BMI, age, fetal sex. P-value <0.05 represented in bold.

Table S5. Association between maternal homocysteine and longitudinal second and third trimester fetal growth in the included population (n= 269), natural pregnancies (n= 145) and IVF/ICSI pregnancies (n= 124).

	Model 1		*Model 2*	
	**BPD**			
	Beta (95%CI) mm	P-value	Beta (95%CI) mm	P-value
Included population n= 243	0.136 (-0.094, 0.367)	0.246	0.088 (-0.144, 0.321)	0.454
Natural pregnancies n= 134	0.188 (-0.774, 0.454)	0.162	0.154 (-0.115, 0.422)	0.257
IVF/ICSI pregnancies n= 109	0.035 (-0.399, 0.470)	0.872	-0.060 (-0.487, 0.366)	0.779
	**HC**			
	Beta (95%CI) mm	P-value	Beta (95%CI) mm	P-value
Included population n= 243	0.341 (-0.186, 0.868)	0.204	0.248 (-0.303, 0.799)	0.375
Natural pregnancies n= 134	0.333 (-0.314, 0.980)	0.309	0.283 (-0.374, 0.938)	0.393
IVF/ICSI pregnancies n= 109	0.205 (-0.817, 1.227)	0.691	0.178 (-0.802, 1.158)	0.719
	**TCD**			
	Beta (95%CI) mm	P-value	Beta (95%CI) mm	P-value
Included population n= 207	0.011 (-0.088, 0.110)	0.832	0.002 (-0.098, 0.104)	0.962
Natural pregnancies n= 119	-0.014 (-0.123, 0.094)	0.796	-0.022 (-0.131, 0.087)	0.688
IVF/ICSI pregnancies n= 88	0.052 (-0.137, 0.241)	0.586	0.025 (-0.168, 0.218)	0.797
	**AC**			
	Beta (95%CI) mm	P-value	Beta (95%CI) mm	P-value
Included population n= 243	0.195 (-0.577, 0.967)	0.619	0.126 (-0.657, 0.909)	0.751
Natural pregnancies n= 134	0.405 (-0.561, 1.372)	0.408	0.394 (-0.591, 1.381)	0.429
IVF/ICSI pregnancies n= 109	-0.238 (-1.555, 1.077)	0.719	-0.477 (-1.811, 0.856)	0.478
	**FL**			
	Beta (95%CI) mm	P-value	Beta (95%CI) mm	P-value
Included population n= 243	-0.046 (-0.201, 0.108)	0.554	-0.093 (-0.250, 0.064)	0.225
Natural pregnancies n= 134	0.092 (-0.101, 0.285)	0.345	0.047 (-0.151, 0.246)	0.635
IVF/ICSI pregnancies n= 109	**-0.336 (-0.590, -0.083)**	**0.009**	**-0.380 (-0.625, -0.134)**	**0.003**
	**EFW**			
	Beta (95%CI) g	P-value	Beta (95%CI) g	P-value
Included population n= 244	1.104 (-8.819, 11.027)	0.826	-0.567 (-10.685, 9.550)	0.912
Natural pregnancies n= 135	6.041 (-6.615, 18.697)	0.346	5.051 (-7.902, 18.003)	0.448
IVF/ICSI pregnancies n= 109	-8.819 (-25,251, 7.611)	0.288	-12.256 (-28.902, 4.389)	0.145

AC= abdominal circumference, BPD= biparietal diameter, EFW= estimated fetal weight, FL= femur length, HC= head circumference; TCD= transcerebellar diameter. Model 1: adjusted for GA, Model 2: adjusted for GA, conception mode (included population only), parity, smoking, folic acid supplement use, alcohol, BMI, age, fetal sex. P-value <0.05 represented in bold.

Table S6. Association between maternal homocysteine and neonatal birth weight in the included population (n= 1060), natural pregnancies (n= 555) and IVF/ICSI pregnancies (n= 505).

	Model 1		*Model 2*	
	**Birth weight**			
	Beta (95%CI) g	P-value	Beta (95%CI) g	P-value
Included population n= 983	-0.486 (-18.124, 17.152)	0.957	-1.857 (-20.121, 16.407)	0.842
Natural pregnancies n= 529	-11.757 (-33.945, 10.431)	0.298	-21.322 (-44.846, 2.201)	0.076
IVF/ICSI pregnancies n= 454	16.878 (-12.193, 45.949)	0.254	24.989 (-4.317, 54.282)	0.094
	**SGA**			
	OR (95%CI)	P-value	OR (95%CI)	P-value
Included population n= 976	0.989 (0.882, 1.109)	0.850	0.968 (0.857, 1.093)	0.600
Natural pregnancies n= 525	1.029 (0.894, 1.184)	0.689	1.011 (0.868, 1.178)	0.886
IVF/ICSI pregnancies n= 451	0.917 (0.754, 1.116)	0.388	0.901 (0.732, 1.110)	0.329
	**LGA**			
	OR (95%CI)	P-value	OR (95%CI)	P-value
Included population n= 976	0.985 (0.860, 1.128)	0.826	0.950 (0.816, 1.105)	0.504
Natural pregnancies n= 525	0.976 (0.828, 1.150)	0.772	0.919 (0.757, 1.115)	0.391
IVF/ICSI pregnancies n= 451	0.984 (0.774, 1.250)	0.893	1.020 (0.796, 1.308)	0.875
	**Hoftiezer percentiles**			
	Beta (95%CI)	P-value	Beta (95%CI)	P-value
Included population n= 976	-0.002 (-0.013, 0.010)	0.770	-0.003 (-0.016, 0.009)	0.587
Natural pregnancies n= 525	-0.010 (-0.025, 0.005)	0.174	**-0.017 (-0.033, 0.000)**	**0.046**
IVF/ICSI pregnancies n= 451	0.012 (-0.007, 0.030)	0.224	0.015 (-0.004, 0.034)	0.131

SGA= small-for-gestational age, LGA=large-for-gestational age. Model 1: adjusted for GA (not applied for hoftiezer percentiles), Model 2: adjusted for GA, conception mode (included population only), parity, smoking, folic acid supplement use, alcohol, BMI, age, fetal sex. P-value <0.05 represented in bold.

Table S7. Association between quartiles of homocysteine and Hoftiezer percentiles in the included population (n= 983), natural pregnancies (n= 529) and IVF/ICSI pregnancies (n= 454).

		Model 1		*Model 2*	
	**Included population**				
		Beta (95%CI)	P-value	Beta (95%CI)	P-value
n= 229	**Q1**	Reference		Reference	
n= 233	**Q2**	-0.038 (-0.091,0.015)	0.159	-0.042 (-0.097,0.014)	0.140
n= 297	**Q3**	-0.010 (-0.035,0.016)	0.455	-0.013 (-0.039,0.013)	0.329
n= 224	**Q4**	-0.006 (-0.024,0.012)	0.507	-0.010 (-0.029,0.009)	0.310
	**Natural pregnancies**				
		Beta (95%CI)	P-value	Beta (95%CI)	P-value
n= 127	**Q1**	Reference		Reference	
n= 111	**Q2**	-0.019 (-0.095,0.056)	0.617	-0.053 (-0.131,0.025)	0.184
n= 155	**Q3**	-0.021 (-0.056,0.014)	0.229	-0.036 (-0.073,0.000)	0.051
n= 136	**Q4**	-0.019 (-0.043,0.005)	0.116	**-0.031 (-0.057,-0.005)**	**0.018**
	**IVF/ICSI pregnancies**				
		Beta (95%CI)	P-value	Beta (95%CI)	P-value
n= 102	**Q1**	Reference		Reference	
n= 122	**Q2**	-0.050 (-0.126,0.025)	0.189	-0.028 (-0.108,0.051)	0.481
n= 142	**Q3**	0.004 (-0.033,0.042)	0.826	0.011 (-0.026,0.048)	0.567
n= 88	**Q4**	0.012 (-0.017,0.041)	0.423	0.016 (-0.014,0.047)	0.287

Model 1: crude, Model 2: adjusted conception mode (included population only), parity, smoking, folic acid supplement use, alcohol, BMI, age, fetal sex. Quartiles of homocysteine are represented as Q1 2.5-5.2 µmol/L, Q2 5.3-6.0 µmol/L, Q3 6.1-7.2 µmol/L, Q4 7.3-14.9 µmol/L, and Q1 was used as reference category. P-value <0.05 represented in bold.
